# Aerosol Jet Printed 3D Electrochemical Sensors for Protein Detection

**DOI:** 10.3390/s18113719

**Published:** 2018-11-01

**Authors:** Edoardo Cantù, Sarah Tonello, Giulia Abate, Daniela Uberti, Emilio Sardini, Mauro Serpelloni

**Affiliations:** 1 Department of Information Engineering, University of Brescia, Via Branze 38, 25123 Brescia, Italy; s.tonello@unibs.it (S.T.); emilio.sardini@unibs.it (E.S.); 2 Department of Molecular and Translational Medicine, University of Brescia, Viale Europa 11, 25123 Brescia, Italy; g.abate001@unibs.it (G.A.); daniela.uberti@unibs.it (D.U.)

**Keywords:** voltammetric sensors, aerosol jet printing, protein detection, 3-D printing

## Abstract

The use of electrochemical sensors for the analysis of biological samples is nowadays widespread and highly demanded from diagnostic and pharmaceutical research, but the reliability and repeatability still remain debated issues. In the expanding field of printed electronics, Aerosol Jet Printing (AJP) appears promising to bring an improvement in resolution, miniaturization, and flexibility. In this paper, the use of AJP is proposed to design and fabricate customized electrochemical sensors in term of geometry, materials and 3D liquid sample confinement, reducing variability in the functionalization process. After an analysis of geometrical, electrical and surface features, the optimal layout has been selected. An electrochemical test has been then performed quantifying Interleukin-8, selected as reference protein, by means of Anodic Stripping Voltammetry. AJP sensors have been compared with standard screen-printed electrodes in terms of current density and relative standard deviation. Results from AJP sensors with Ag-based Anodic Stripping Voltammetry confirmed nanostructures capability to reduce the limit of detection (from 2.1 to 0.3 ng/mL). Furthermore, AJP appeared to bring an improvement in term of relative standard deviation from 50 to 10%, if compared to screen-printed sensors. This is promising to improve reliability and repeatability of measurement techniques integrable in several biotechnological applications.

## 1. Introduction

Recently, printed electronics have been increasingly investigated as convenient and promising for providing reliable feedbacks on biological samples or physiological processes, in applications ranging from diagnostics, pharmaceutics to tissue engineering. Moreover, the recent attention for disposable, low-cost and reliable biomolecule-to-chip interface systems for high-throughput in-vitro assays is becoming an urgent need due to novel international regulatory guidelines [1].

Nowadays the techniques adopted most frequently for these applications are screen printing (SP) and ink-jet printing (IJP). They both allow achieving resolution up to 50–100 µm, required to provide proper geometrical properties electrochemical sensors for a wide range of biotechnological applications such as chemicals detection, DNA or protein recognition [2,3].

The feasibility of SP for flexible electronics has been demonstrated through a number of printed sensors, electronics devices and circuits [4]. Regarding the area of biotechnological applications, the most used and accepted design is the one commercially available produced by companies such as Dropsens or Metrohm, which provide a very wide variety of different materials or designs, easily manageable and applicable to different areas of biotechnological research [5].

IJP is another technique rapidly emerging for biotechnological applications. Its main advantage, compared to SP, is the possibility of direct patterning solution based materials with a maskless procedure [6].

Despite the cost and time effectiveness of both these techniques, they present some issues in term of reproducibility, resolution, and difficulty to realize 3D structures useful for a proper management of liquid samples. Several articles have been dedicated to evaluate and compare the performances of different material and sensors producers and electrochemical techniques [5]. Regarding SP, solution viscosity, printing speed, angle and geometry of the squeegee, distance between screen and substrate, mesh size and material represent critical factors that can strongly influence the final device [7]. The paste viscosity and surface tension of the substrate might thus limit the available substrates through the mask depending on their surface chemistry.

IJP as well, despite clear advantages due to mask-free processing, presents the main challenges related to slow speed due to the limited number of nozzles and possible clogging, challenging when addressing an industrial production. Finally, other difficulties are related with the limited range of viscosities and the limited control of shape, thickness and morphology of the dried ink due to the variability of droplets spreading [8,9].

In this picture, Additive Manufacturing (AM) might represent a promising technique to combine an improved resolution, customization, and standardization. The possibility to scale-up the realization of sensors combining electrodes with 3D structures might allow the production of novel biosensors, integrating bioelectronics with a suitable 3D environment for biological assays [10,11]. Among the extremely various techniques available in AM, Aerosol Jet Printing (AJP) represents one of the newest and most promising in term of reproducibility and high resolution [12]. AJP is a non-contact printing technique, in the family of droplet-based direct-write (known also as M3D, maskless mesoscale materials deposition), developed by Optomec (Albuquerque, NM, USA) under the Defense Advanced Research Projects Agency (DARPA) Mesoscopic Integrated Conformal Electronics (MICE) program. This technique works by atomizing a solution/suspension containing the functional material, which is deposited onto a substrate placed on a heatable plate to realize specific surface features (e.g., dots or lines). Once the mist is generated inside the atomizer, it passes through the virtual impactor to regulate the droplets’ dimensions (smaller than 5 μm in diameter) using as pivotal parameter particles’ momentum: particles with a diameter greater than 5 μm collide with the walls and are collected in the filter, those too small do not enter the impactor and collapse inside the atomizer. Droplet diameter sizes from 1–5 μm offer a unique uniform printing dispersion respect to the other techniques. Then the aerosol is transported to the printing head where a secondary gas flow cylindrically envelops the aerosol and focuses it on the specific substrate [13,14,15]. Compared with traditional printing electronics techniques, this process ensures high performances and customization, allowing to print traces from 10 μm to 5 mm in width, without the use of masks or post-patterning and with a wide range of inks suitable for advanced applications ranging from energy harvesting and flexible electronics to devices for bio-electronics applications [16]. More in details, one of AJP major benefit is related to ink viscosity. Differently from the limited ranges of viscosities suitable for IJP (5–20 cP) and for SP (>1000 cP), AJP allows the usage of inks in the viscosity range of 1–1000 cP, permitting to employ and combine a broader range of functional materials (metals, insulators, ceramics, semiconductors, polymers, biological material) [17,18]. The final results achievable with AJP are related to atomization properties and parameters, the modulation of these parameters introduces a higher possibility of customization respect to SP and IJP [19]. Furthermore, since AJP is a mist-generating technique, it could ensure a higher depositing speed respect to drop-on-demand techniques. In particular, focusing on the comparison between AJP and IJP, despite AJP can theoretically print at higher velocity, generally in the AJP literature printing velocity tends to be in the order of few mm/s to achieve a reasonable line thickness [17]. Among the direct printing techniques, AJP is the only one capable to print and focus the printing stream on irregular 3D surfaces and complex geometries (e.g., bio-functionalization of prostheses) and to precisely patterning microarrays sensors (e.g., with specific enzymes or proteins) [20,21]. This is possible due to the lower amount of ink required by AJP, thanks to the usage of ultrasonic atomizer which requires a very small amount of ink (less than 1 mL, comparable with IJP), due to the low temperatures and to the low dispersion of the printed droplets particularly interesting when dealing with expensive biological material like antibodies, enzyme or protein [18,21,22]. The focusing system of AJP, together with tunable process parameters, is also responsible for the high resolution of this technique and to avoid possible clogging of nozzles, which are drawbacks respectively for SP and IJP. Finally, AJP allows multi-materials and multilayer printing designs, which ensure flexibility, scalability and the possibility to print multifunctional features if compared to traditional technologies [23]. Furthermore, AJP shows the potential to enhance its current printing resolution, which is already high respect to IJP and SP, aiming to be the most suitable technology for additive manufacturing of high-quality and high-performance electrical components in near future [18].

Focusing on the current trends of electrochemical sensors applied in medicine, biotechnology, and pharmacology where high levels of standardization, repeatability, sensitivity, and miniaturization are demanded [24], the potential of AJP in term of reproducibility, resolution and 3D customization represents a valuable source [11,25]. The most significant applications range from pH and ion sensing to cell monitoring and protein detection. Regarding pH sensing, sensors with high-resolution tracks (20 µm) have been fabricated with AJP in [26] using carbon nanotubes ink in order to combine low-cost and flexibility with sensitivity. Sensors demonstrated high repeatability, fast response time and excellent biocompatibility required for live cells. Another interesting application described in [11] address cell potential monitoring: silver microelectrode arrays (MEA) with customized electrode spacing were fabricated using AJP technology, giving a fundamental starting point for low-cost custom-shaped MEA customizable for a wide range of electrochemical platforms. Regarding electrochemical detection of protein using printed electronics, the main trends in the most updated research refers to the attempt to introduce nanomaterials, such as carbon nanotubes [27], to improve sensitivity, new techniques such as imprinted polymers or specific functionalization to improve the selectivity [28] and high resolution printing techniques to improve the control over reproducibility [21]. Considering these interests, exploiting the potential of AJP, promising results have been demonstrated by directly patterning sensors with micro-arrays of small proteins and biomolecules using the very same set up described, with promising results for those specific application requiring a very precise positioning of the bio-molecules or requiring a single bio-coating to produce a ready-to-use device [21]. Finally, another use of AJP technique to improve electrochemical sensor performances can be found in [29], where AJP optimal control over thickness and lateral resolution could allow achieving a higher sensitivity by realizing a CNTs-based coating AJ printed over SPEs.

In this picture, AJP is here investigated as promising tool for improving the requirements of electrochemical sensors, in term of both repeatability and limit of detection (LOD), thanks to an optimization of the biofunctionalization process of the working electrode (WE), achieved ensuring a proper liquid confinement by a customized 3D environment, which avoids possible leakages while managing liquid samples. Microscopy glass slides with a sample holding concavity were chosen as substrates, in order to realize a device easily integrable with the laboratory routine. The ability of AJP to print also on these curve non-porous substrates makes possible from one side to combine electrical measurements with optical or biochemical assays and from the other side makes simpler to manage liquid samples in the concavity. This specific attention to confine liquid has been also ensured exploiting the potential of AJP as AM technique, by depositing a UV curable material (NOA) to realize a 3D structure similar to a real well. In light of this, the present work proposes the use of AJP to realize a customized measuring device with electrochemical sensors, addressable for the analysis of biological samples for protein quantification.

After a detailed description of the materials and the methods followed for device fabrication and features testing, the validation of the AJP sensors printed on glass slides is presented using Interleukin-8 (IL-8) chosen as reference protein, due to its multiple applications in diagnostics and biotechnological research. The high number of replicates performed for each concentration, allowed to perform critical considerations from the metrological point of view, comparing AJP results with the ones obtained from screen-printed electrodes (SPEs).

## 2. Materials and Methods

### 2.1. Sensors Design and Material Choice

**Figure 1:**
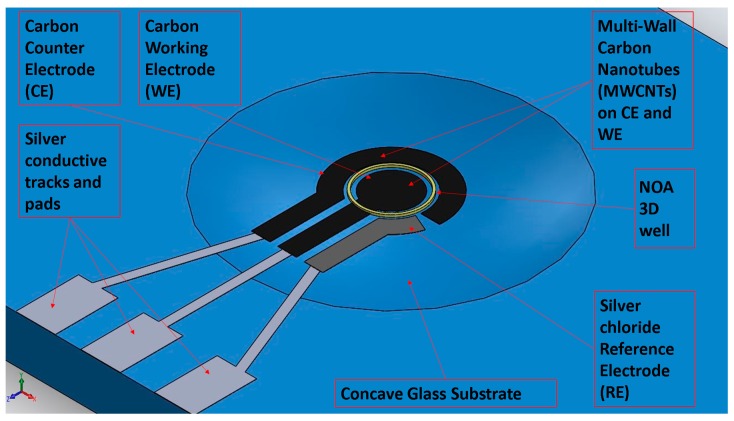
Schematic representation of the final prototype.

After evaluating available designs on the market and in the literature [30], a 3-electrodes system (working (WE), counter (CE) and reference (RE) electrodes), usually employed for electrochemical measurements, was selected as layout (Figure 1). 

In order to realize proper sensing devices suitable for microscopy and for easy managing during biological sample modifications, concave glass slides were selected as substrates. In addition to a proper rigidity, glass provides an optimal transparency and suitable dimensions standardized for most of the instrumentation used for biochemical and optical assays. Figure 1 shows an example of all the layers corresponding to the different employed inks: (i) silver for the conductive tracks, (ii) carbon for WE and CE, (iii) silver chloride for RE and (iv) NOA 81 for creating a sort of delimiting hedge for liquid samples. Further, an electronic performances improvement and an increase of the surface to volume ratio for biofunctionalization have been achieved by printing an additional layer of MWCNTs (multiwall carbon nanotubes) over carbon electrodes. Silver ink is produced by UTDots Inc. (Champaign, IL, USA), with its own thinner, UTDAg ink is based on silver nanoparticles with an average size around 10 nm and dispersed in a liquid vehicle. Nanosilver concentration is about 25–60 wt.%, with a viscosity of 1–30 cP. Since they are surface stabilized, UTDAg inks are highly soluble in nonpolar organic solvents and stable under atmospheric conditions at room temperature. Silver chloride ink (XA-3773) was purchased from Fujikura Kasei. Co. Ltd. (Shibakouen Minato-ku, Tokyo, Japan) together with its own thinner. The ink was chosen with Ag/AgCl weight proportion ratio of 8/2. Since the ink starting viscosity was 300 ± 50 dPa∙s, it was necessary to dilute the ink with its thinner before printing, following the equations present in the literature regarding a two-component blend [31]. Carbon ink (EXP 2652-28) characterized by a starting viscosity of 15–20 Pa∙s was purchased from Creative Materials Inc. (Ayer, MA, USA). The layer of MWCNTs was obtained printing Nink 1000, commercialized by NANOLAB (Waltham, MA, USA). It is a carbon nanotube ink for direct printing techniques containing carboxyl (–COOH) functionalized carbon nanotubes in an aqueous suspension (the viscosity is proximal to the one of water) with the minimum concentration of additives to impart long-term stability and printability to the ink. Finally, the UV-curable polymer NOA 81 exploits the ability of the AJ printer to realize a 3D structure able to confine the liquid sample only on WE, as needed during specific steps of sensors functionalization. NOA 81 was purchased from Norland Products (Cranbury, NJ, USA). It is a fast UV-curing adhesive, which produces, after curing, a hard, resilient bond. The material is characterized by a viscosity of 300 cP at 25 °C, showing an excellent adhesion on glass and metal, and a fair adhesion on plastics. The different process parameters are summarized in Table 1. Each ink was printed with two consecutive depositions, followed by its own specific heat treatment, using a 200 μm nozzle tip. Pneumatic atomization was selected. The final line width was about 60 μm.

### 2.2. Sensor Fabrication

**Figure 2:**
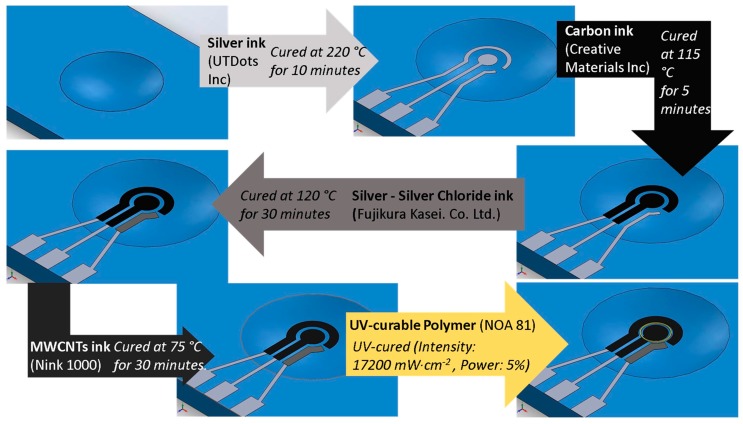
AJP process for sensor fabrication.

An aerosol jet printer (AJ300) commercialized by Optomec was used in order to fabricate all the electrochemical sensors. Each conductive layer has been printed and cured following the sequence and the curing parameters reported in Figure 2. In the last step, a hollow cylinder of around 30 µm height was obtained by depositing a 3D spiral composed by 5 circles, to achieve a structure able to contain samples up to 20 µL but not limiting the possibility to perform the measurements using the three electrodes structure. In order to control precisely the height of the 3D customized walls, NOA 81 has been UV-cured right after deposition using as UV curing system the LED Spot type Panasonic ANUJ6180 series, model 6423 (Panasonic, Kadoma, Osaka, Japan) characterized by a spot diameter of 3 mm, a peak UV intensity of 17,200 mW∙cm^−2^ at an irradiation distance of 8 mm. In our experimentation, the select power was 5% of the peak intensity. 

**Figure 3:**
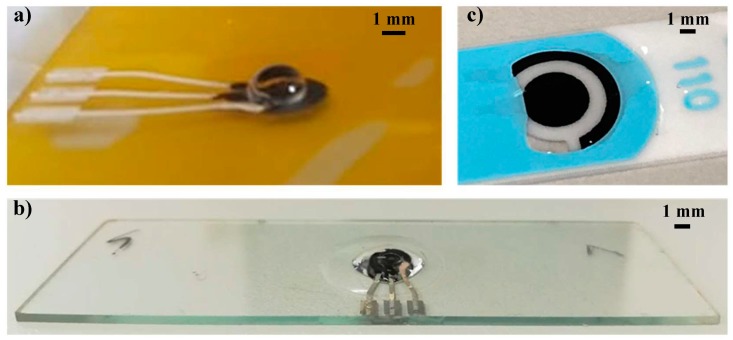
(**a**) Final layout of the printed sensorized glass slide with sample confinement during WE functionalization; (**b**) Example of the sample confinement over the three electrodes during measurement; (**c**) Example of liquid leakage on a commercial screen-printed sensor.

These sensors were fabricated in different sizes, in order to evaluate the correlation between the miniaturization of electrochemical sensors and their performances in term of repeatability, sensitivity, and LOD. The initial comparison among the different geometries has been performed fabricating 10 sensors for each geometry. The selected geometry has been then replicated fabricating other 10 sensors with the same geometry. In Figure 3a, a drop of a buffer solution has been deposited on the WE electrode highlighting the ability of the NOA ring to efficiently contain liquid sample during functionalization, avoiding any leakage often experienced with SPEs (Figure 3b).

### 2.3. Geometrical Analysis and Electrical Resistances

In order to assess the suitability of substrate-ink combination to test liquid biological samples, different aspects of the sensors were investigated. After analyzing geometrical and electrical parameters of the printed tracks, the effective coating of a layer of antibodies on the surface of AJP sensors was assessed using fluorescence imaging.

Regarding geometrical analysis, a diamond stylus-based system for step height measurements, (Alpha-Step IQ Kla Tencor profilometer, Kla-Tencor, Milpitas, CA, USA), has been used to measure printed layers thickness, with a range 8 nm–2 mm and an uncertainty of 0.1%.

Electrical resistance was then evaluated using a Hewlett-Packard 34401a digital bench-top multimeter (HP, Palo Alto, CA, USA), applying testing probes to the extremities of each path, in standardized and repeatable points, thus measuring the resistance offered by all its length. Each measure has been repeated ten times, in order to ensure the proper calculation of the mean values and of the standard deviations.

Resistivity was then calculated from the classical equation R = ρ∙l∙S^−1^ where R is resistance, ρ is resistivity, l is the length of the considered path and S its section.

After evaluating geometrical and electrical features, the possibility to functionalize WE with an effective protein coating has been assessed using fluorescence imaging with a near infrared imaging system. More specifically, the binding between carbon WE and the primary antibody anti-human IL-8 (8 µg/mL), produced in mouse (Duo Set kit), was evaluated recording the emitted light deriving from a secondary antibody specie-specific (anti-mouse), labelled with a fluorescent tag and its signal was acquired with Odyssey^®^ Fc Dual-Mode Imaging System from LI-COR Biosciences (Lincoln, NE, USA). Thus, sensors were washed and excited with a 685 nm light source to study coating deposition, keeping two electrodes as control blank samples (i.e., covered with a buffer solution of phosphate buffered saline). 

### 2.4. Voltammetry-Based Protein Quantification

The protocol adopted for testing sensors’ ability to quantify proteins involved IL-8 as reference protein for the assay. IL-8 quantification was characterized by a specific functionalization of the sensor using immunocomplexes formed by a capture and a detection antibody (DuoSet ELISA kit, Human CXCL8/IL-8, R&D System, Minneapolis, MN, USA), and by an Anodic Stripping Voltammetry (ASV) based measurement technique, optimized in [32,33].

**Figure 4:**
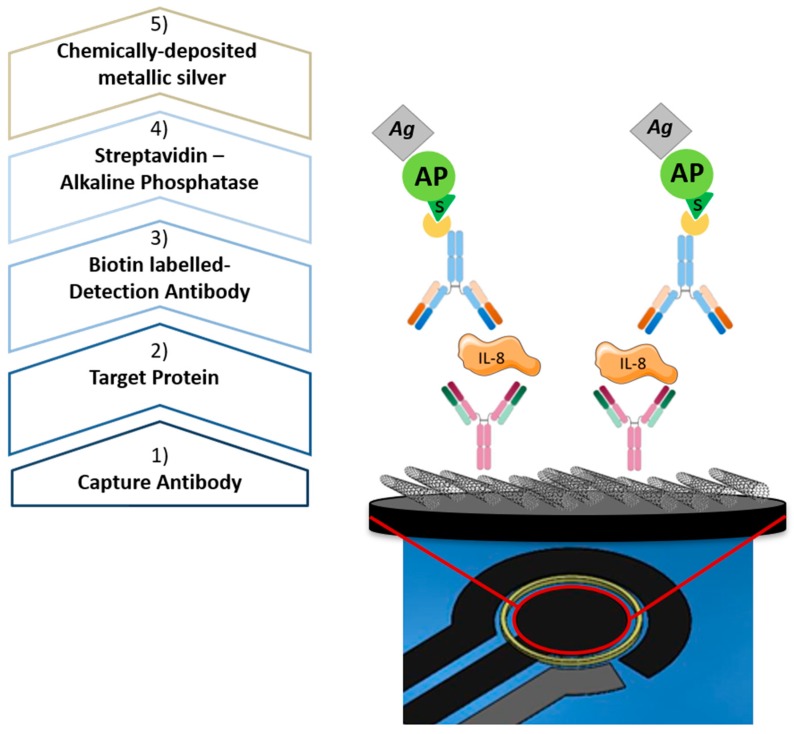
Bio-functionalization protocol for the ASV measurements of IL-8.

In detail, each WE was exposed to the following bio-functionalization steps (Figure 4): (i) overnight immobilization of anti-IL-8 antibody to sensor surfaces via drop-casting; (ii) 2-h incubation with recombinant human IL-8, at different concentrations; (iii) 2 h incubation with biotin-labelled detection antibody; (iv) addition of streptavidin-tagged alkaline phosphatase (AP) enzyme that catalyzes the oxidation of ionic Ag (AgNO_3_) to metallic Ag, thanks to the reaction happening in presence of ascorbic acid (AA-p), as described in [34]. Once completing the bio-functionalization, sensors were covered with PBS in order to perform the final measurement. A constant potential of −0.12 V was applied for 10 s and then a Linear Sweep Voltammetry (LSV) was performed at a scan rate of 40 mV/s up to +0.4 V, measuring Ag oxidation current. Since the measurement technique allows only one repetition for each sensor, the reproducibility has been tested by testing the same protocol for each selected concentration on ten different sensors for each geometry. All measurements were performed using a potentiostat (Palmsens, Compact Electrochemical Interfaces, Houten, Utrecht, The Netherlands) controlled using a dedicated software (PS Trace 5.3), used to quantify the current peaks corresponding to each concentration and to derive the calibration curve. The LOD has been then calculated using the 3-sigma rule [35,36].

## 3. Results and Discussion

### 3.1. Sensors Testing

Results obtained from the geometrical analysis (Table 2) shows a different thickness for each layer. The different values can be addressed to the different process parameters, number of printed layers and viscosity (higher thicknesses were obtained for inks with higher viscosity) adopted for each ink. The standard deviation is always less than 20% demonstrating an acceptable process variability in the thicknesses for all the printed sensors.

Results from electrical tests show resistance and resistivity data in agreement with the nominal values of the manufacturers, considering the specific process parameters for each ink. Ag experimental resistivity (12.2 × 10^−8 ^Ω∙m) is comparable with the nominal one (3 × 10^−8 ^Ω∙m). The use of the thinner in order to achieve the proper final viscosity may have affected AgCl experimental resistivity value (71.3 × 10^−8 ^Ω∙m), but it can be compared with the nominal one reported by Fujikura Kasei. Co. Ltd. (56 × 10^−8 ^Ω∙m). Finally, C experimental resistivity (10.3 × 10^−4 ^Ω∙m) was slightly decreased compared to the one given by Creative Materials (25 × 10^−4 ^Ω∙m), due to the choice performed during the heat treatment in term of duration and temperature. 

**Figure 5:**
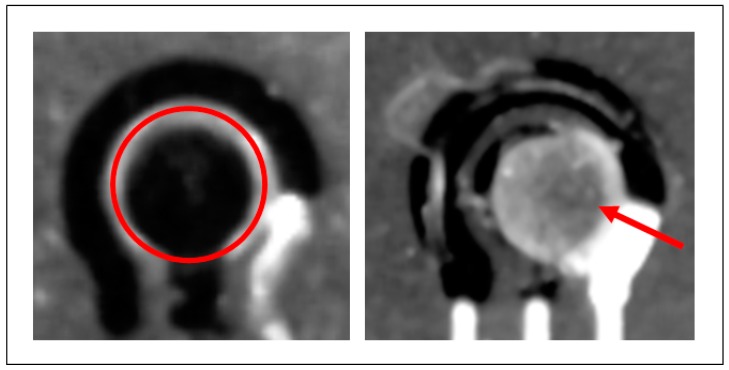
Fluorescence imaging, with grayscale color filter, of a bare carbon working electrode without antibodies (**left**) and with antibodies attached (**right**).

Results obtained from fluorescence imaging shows a clear difference between blank and antibodies coated electrodes (Figure 5). The red arrows indicate the surfaces successfully covered by immunocomplexes, which appear as the only ones emitting in the near infra-red region. Differently, the blank sample, treated with the same protocol, but incubated with PBS instead of IL-8 recombinant protein, do not emit any light, confirming the specificity of the binding between protein and immunocomplexes.

Thanks to these confirmations, it is possible to state that these materials and AJP technique can be employed to produce electrodes allowing a homogeneous adhesion of antibodies, essential to perform a complete functionalization to perform immune-sensing of proteins.

### 3.2. Voltammetry-Based Protein Quantification

**Figure 6:**
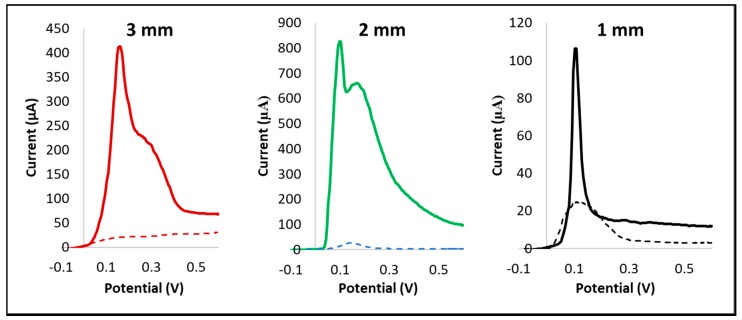
Plots obtained during protein quantification test; each plot measures current (expressed in μA) as a function of potential (expressed in V). Dotted lines represent “blank samples”.

IL-8 was selected as the protein to be tested to evaluate the possibility of using the sensor designed for voltammetric based protein quantification. This member of the CXC chemokine subfamily is considered as universal biomarkers—from cancer and inflammation to neurodegeneration [37]—thus making the present PoC platform available for various applications in the clinical field. Furthermore, IL-8 strong interaction with its capture antibody allows reducing the variability to the functionalization phase. Results in Figure 6 report the comparison performed among the three different geometries in quantifying 10 ng/mL of IL-8 sample using ASV.

All the peaks obtained in the ASV has been quantified and summarized in Table 3, both in term of current and of current density, normalized for the WE area, averaged among ten replicates. For all the evaluated geometries, a clear difference in peaks heights could be appreciated between blank and samples (Figure 6), confirming the possibility to successfully apply the described ASV protocol for protein quantification on all the AJP sensors.

In order to better discuss these results, it is fundamental to take into account both electrical and biological point of view. 3 mm sensors, with a geometry similar to the commercial SPEs produced by Dropsens, despite presenting a quite high absolute current peak of 281 μA, does not appear as the most performing one in term of current density (39.7 μA/mm^2^) (Table 3). 1 mm sensors, instead, despite a clearly reduced height of the absolute current peak due to the limited area for electrodes exchange, present a higher current density of about 104.5 μA/mm^2^ compared with 3 mm sensors, and showed really sharp peaks with a very low background noise. 

Interestingly, the results obtained for the 2 mm WE appeared as the most performing one, especially in term of current density, 201.3 μA/mm^2^. This result can be explained with the closeness between WE and CE obtained shrinking electrochemical cell dimensions, which appear to be significative to enhance the current peak height and reduce the current loss and the background noise. These features, combined with the compatibility of the dimensions with a manual functionalization, make this geometry the most suitable to reduce sample volume, without sacrificing performances and sensitivity. These results allowed selecting the 2 mm geometry as the most suitable ones for a complete test analysis selected in a range of concentration from 1.25 to 10 ng/mL of IL-8 solution.

**Figure 7:**
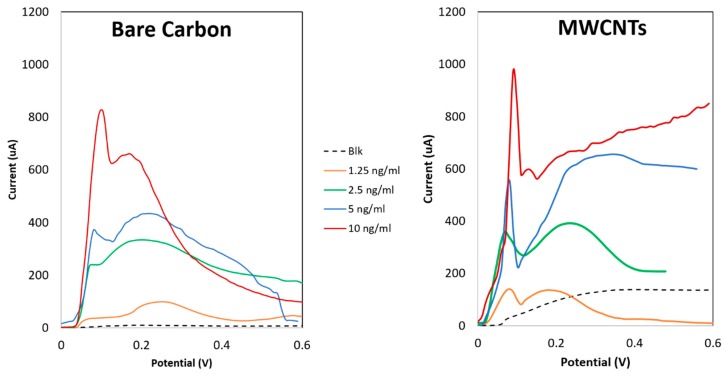
LSV for IL8 quantification performed using bare and nanostructured carbon-based sensors.

Figure 7 and Table 4 summarize the results obtained performing the analysis with the ASV protocol on two types of printed sensors, on bare C electrodes and on MWCNTs functionalized WE. Considering the curves in both the plots in Figure 7, referring to AJP sensors, clear oxidative peaks could be observed around a potential of 0.1 V, corresponding to the Ag stripping from WE. A slight potential shift (around 10 mV) could be observed in the MWCNTs WE compared to the bare C ones, suggesting an improved electron transfer due to the nanostructures, making easier the oxidation of the Ag. Furthermore, comparing the curves of bare C with MWCNTs in Figure 7 and LOD of Table 4, it can be highlighted how the nanostructures decrease the LOD of 7 folds, in agreement with what could be observed in previous works performed with SPEs [33].

Additionally, the performance of AJPEs (Figure 7) has been compared to the one of commercially available C SPEs, both bare and nanostructured, performing ASV with the same protocol (Table 4). 

All the results were obtained from ten replicates for each concentration, in order to perform a critical evaluation of the variability. Due to the different dimensions of the WE, results were compared in term of current density peaks and in term of relative standard deviation (RSD), as the ratio between the standard deviation of ten repetitions and the average value of the current density of each peak.

**Figure 8:**
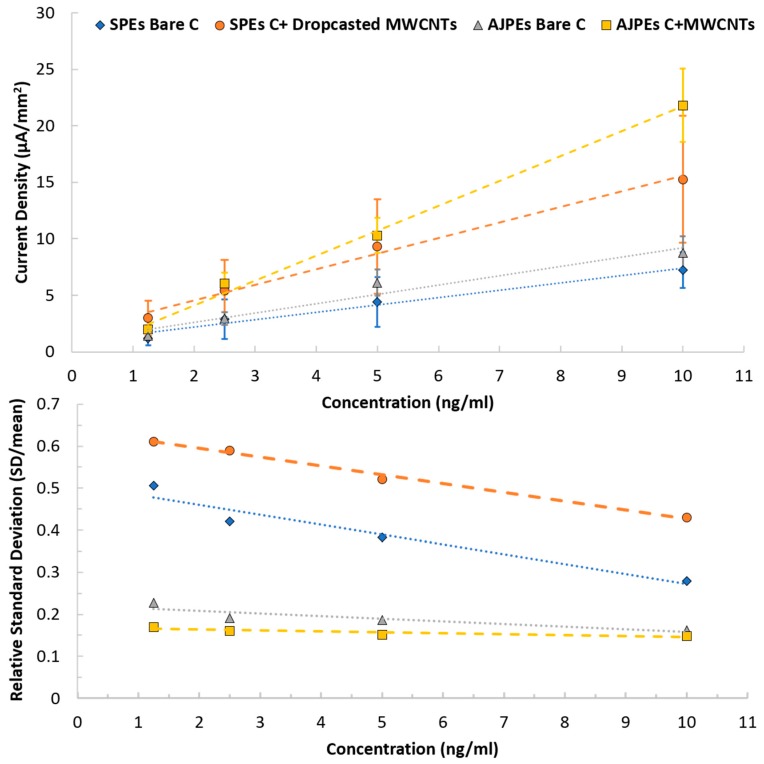
Calibration plot comparison between Carbon bare and nanostructured sensors.

An improvement in term of measurements repeatability, which in turn resulted in a reduced LOD, can be highlighted both from the results in Table 4 than discussing the plots of Figure 8.

Interestingly, the high geometrical resolution and the possibility to control precisely the parameters using AJP appeared to decrease the LOD from 3.4 to 2.1 ng/mL, even when using the same material than in the SP (carbon), with a reduction of the RSD of almost 2 times. The higher control on deposition of the nanostructures appears to have a strong influence as well, considering the decrease of the LOD of almost 2 folds, confirmed by the high reduction of the RSD obtained (from 60% to 15%) comparing drop-casted MWCNTs with AJP ones. Additionally, a significant role that needs to be considered and that might have an influence in reducing variability can be addressed to the presence of the 3D well in the AJPEs, and not in the SPEs, which ensured a proper coating, avoiding unspecific protein deposition on CE or RE.

## 4. Conclusions

In this paper, we have reported the realization of miniaturized electrochemical platforms for protein detection, developed through aerosol jet printing. Electrodes and conductive tracks with a resolution of about ±6 μm and controllable thickness with a standard deviation always less than 20% could be realized using this technique, showing proper values of electrical resistivity coherent with what declared by the manufacturers. Furthermore, antibodies have shown good adhesion to the sensors. Beside confirming the significant improvement of AJP nanostructures compared to AJP bulk material in term of LOD (from 2.1 to 0.3 ng/mL), the obtained results allowed to improve the repeatability, if compared with commercially available screen-printed electrodes, thanks to the better liquid confinement and the lower operator dependency achieved in the functionalization phase. In detail, the RSD could be reduced from 60 to 15%, comparing drop-casted nanostructures to AJP nanostructures, and from 40% to 20%, comparing SP to AJP carbon. The improved repeatability will allow in future studies to deepen the investigation of further metrological characteristics (e.g., resolution) and the possibility to directly AJ print biomolecules (e.g., enzymes, antibodies) to realize ready to use electrochemical sensors. In addition, the validation of NOA as UV-curable printable polymer represents an important starting point to address future works integrating not only a polymer well but a proper microfluidic. Overall, the results obtained suggest this technique as a really promising one to combine reliability and repeatability with the wide possibilities offered by these printed devices for several biotechnological applications.

## Figures and Tables

**Table 1:** Printing process parameters.

Process Parameters	Ag	AgCl	C	NOA 81	Nink 1000
Sheath gas flow (SCCM)	20	30	110	80	65
Exhaust flow (SCCM)	570	570	1000	1400	800
Atomizer flow (SCCM)	550	530	900	1360	750
Process speed (mm s^−1^)	2	2	2	0.75	3.5
Plate temperature (°C)	60	65	70	/	45

**Table 2:** Thickness and sections of deposited inks.

Material	Thickness (μm)	Standard Deviation (μm)	Section (μm^2^)
Ag	6.8	±1	854.2
AgCl	4	±0.8	392.3
C + MWCNTs	6.5	±0.2	365.3
NOA 81	25	±3	1400

**Table 3:** Current peaks heights for 10 ng/mL protein quantification on the glass-substrate sensor.

WE Diameter	Current Peaks (μA)	Standard Deviation (μA)	Current Density (μA/mm^2^)
3 mm	280.8	118.8	39.7
2 mm	632.0	142.0	201.3
1 mm	82.0	27.9	104.5

**Table 4:** LOD obtained from the different conditions, considering different WE materials and printing methods.

	SPEs	AJPEs
Bare C	3.4 ± 0.5 ng/mL	2.1 ± 0.2 ng/mL
MWCNTs	0.5 ± 0.4 ng/mL	0.3 ± 0.2 ng/mL

## References

[1] Spanu A., Lai S., Cosseddu P., Tedesco M., Martinoia S., Bonfiglio A. (2015). An organic transistor-based system for reference-less electrophysiological monitoring of excitable cells. Sci. Rep..

[2] Couto R.A.S., Lima J.L.F.C., Quinaz M.B. (2016). Recent developments, characteristics and potential applications of screen-printed electrodes in pharmaceutical and biological analysis. Talanta.

[3] Li J., Rossignol F., Macdonald J. (2015). Inkjet printing for biosensor fabrication: Combining chemistry and technology for advanced manufacturing. Lab Chip.

[4] Khan S., Lorenzelli L., Dahiya R.S. (2015). Technologies for printing sensors and electronics over large flexible substrates: A review. IEEE Sens. J..

[5] Fanjul-Bolado P., Hernández-Santos D., Lamas-Ardisana P.J., Martín-Pernía A., Costa-García A. (2008). Electrochemical characterization of screen-printed and conventional carbon paste electrodes. Electrochim. Acta.

[6] Jayasinghe S.N., Townsend-Nicholson A. (2006). Bio-electrosprays: The next generation of electrified jets. Biotechnol. J..

[7] Jabbour G.E., Radspinner R., Peyghambarian N. (2001). Screen printing for the fabrication of organic light-emitting devices. IEEE J. Sel. Top. Quantum Electron..

[8] Singh M., Haverinen H.M., Dhagat P., Jabbour G.E. (2010). Inkjet Printing—Process and Its Applications. Adv. Mater..

[9] Kang B.J., Lee C.K., Oh J.H. (2012). All-inkjet-printed electrical components and circuit fabrication on a plastic substrate. Microelectron. Eng..

[10] Ragones H., Schreiber D., Inberg A., Berkh O., Kósa G., Freeman A., Shacham-Diamand Y. (2015). Disposable electrochemical sensor prepared using 3D printing for cell and tissue diagnostics. Sens. Actuators B Chem..

[11] Yang H., Rahman T., Du D., Panat R., Lin Y. (2016). 3-D Printed Adjustable Microelectrode Arrays for Electrochemical Sensing and Biosensing. Sens. Actuators. B Chem..

[12] Secor E.B. (2018). Principles of aerosol jet printing. Flex. Print. Electron..

[13] Hoey J.M., Lutfurakhmanov A., Schulz D.L., Akhatov I.S. (2012). A review on aerosol-based direct-write and its applications for microelectronics. J. Nanotechnol..

[14] Binder S., Glatthaar M., Rädlein E. (2014). Analytical investigation of aerosol jet printing. Aerosol Sci. Technol..

[15] Clifford B., Beynon D., Phillips C., Deganello D. (2018). Printed-Sensor-on-Chip devices—Aerosol jet deposition of thin film relative humidity sensors onto packaged integrated circuits. Sens. Actuators B Chem..

[16] OPTOMEC (2018). Aerosol Jet ® Printed Electronics Overview.

[17] Seifert T., Sowade E., Roscher F., Wiemer M., Gessner T., Baumann R.R. (2015). Additive manufacturing technologies compared: Morphology of deposits of silver ink using inkjet and aerosol jet printing. Ind. Eng. Chem. Res..

[18] Tan H.W., Tran T., Chua C.K. (2016). A review of printed passive electronic components through fully additive manufacturing methods. Virtual Phys. Prototyp..

[19] Agarwala S., Goh G.L., Yeong W.Y. (2017). Optimizing aerosol jet printing process of silver ink for printed electronics. IOP Conf. Ser. Mater. Sci. Eng..

[20] Paulsen J., Renn M., Christenson K., Plourde R. Printing conformal electronics on 3D structures with Aerosol Jet technology. Proceedings of the FIIW 2012—2012 Future of Instrumentation International Workshop Proceedings.

[21] Grunwald I., Groth E., Wirth I., Schumacher J., Maiwald M., Zoellmer V., Busse M. (2010). Surface biofunctionalization and production of miniaturized sensor structures using aerosol printing technologies. Biofabrication.

[22] Narayan R. (2014). Rapid Prototyping of Biomaterials: Principles and Applications.

[23] Smith M., Choi Y.S. Optimizing aerosol jet printing process of silver ink for printed electronics Optimizing aerosol jet printing process of silver ink for printed electronics. Proceedings of the IOP Conference Series: Materials Science and Engineering.

[24] Da Silva Neves M.M., González-García M.B., Hernández-Santos D., Fanjul-Bolado P. (2018). Future trends in the market for electrochemical biosensing. Curr. Opin. Electrochem..

[25] Agarwal K., Hwang S., Bartnik A., Buchele N., Mishra A., Cho J.-H. (2018). Small-Scale Biological and Artificial Multidimensional Sensors for 3D Sensing. Small.

[26] Goh G.L., Agarwala S., Tan Y.J., Yeong W.Y. (2018). A low cost and flexible carbon nanotube pH sensor fabricated using aerosol jet technology for live cell applications. Sens. Actuators B Chem..

[27] Landry M.P., Ando H., Chen A.Y., Cao J., Kottadiel V.I., Chio L., Yang D., Dong J., Lu T.K., Strano M.S. (2017). Single-molecule detection of protein efflux from microorganisms using fluorescent single-walled carbon nanotube sensor arrays. Nat. Nanotechnol..

[28] Ma Y., Shen X.-L., Zeng Q., Wang H.-S., Wang L.-S. (2017). A multi-walled carbon nanotubes based molecularly imprinted polymers electrochemical sensor for the sensitive determination of HIV-p24. Talanta.

[29] Kuberský P., Altšmíd J., Hamáček A., Nešpůrek S., Zmeškal O. (2015). An Electrochemical NO_2_ Sensor Based on Ionic Liquid: Influence of the Morphology of the Polymer Electrolyte on Sensor Sensitivity. Sensors.

[30] Gong C.S.A., Syu W.J., Lei K.F., Hwang Y.S. (2016). Development of a flexible non-metal electrode for cell stimulation and recording. Sensors.

[31] Zhmud B. (2014). Lube-Tech093-ViscosityBlendingEquations.

[32] Tonello S., Abate G., Borghetti M., Marziano M., Serpelloni M., Uberti D.L., Lopomo N.F., Memo M., Sardini E. (2017). Wireless Point-of-Care Platform with Screen-Printed Sensors for Biomarkers Detection. IEEE Trans. Instrum. Meas..

[33] Tonello D.U.S., Marziano M., Abate G., Kilic T., Memo M., Carrara E.S.S., Lopomo N.F., Serpelloni M. Enhanced Sensing of Interleukin 8 by Stripping Voltammetry: Carbon Nanotubes versus Fullerene. Proceedings of the European Medical and Biological Engineering Confernce.

[34] Chen Z.P., Peng Z.F., Jiang J.H., Zhang X.B., Shen G.L., Yu R.Q. (2008). An electrochemical amplification immunoassay using biocatalytic metal deposition coupled with anodic stripping voltammetric detection. Sens. Actuators B Chem..

[35] Shrivastava A., Gupta V. (2011). Methods for the determination of limit of detection and limit of quantitation of the analytical methods. Chron. Young Sci..

[36] Armbruster D.A., Pry T. (2008). Limit of blank, limit of detection and limit of quantitation. Clin. Biochem. Rev..

[37] Kim H.J., Li H., Collins J.J., Ingber D.E., Beebe D.J., Ismagilov R.F. (2016). Contributions of microbiome and mechanical deformation to intestinal bacterial overgrowth and inflammation in a human gut-on-a-chip. Proc. Natl. Acad. Sci. USA.

